# Mutation and Diversity of Diphtheria Toxin in *Corynebacterium ulcerans*

**DOI:** 10.3201/eid2511.181455

**Published:** 2019-11

**Authors:** Ken Otsuji, Kazumasa Fukuda, Midori Ogawa, Mitsumasa Saito

**Affiliations:** University of Occupational and Environmental Health Japan, Kitakyushu, Japan

**Keywords:** Diphtheria toxin, *Corynebacterium ulcerans*, diversity, amino acid sequence, bacteria, Japan

## Abstract

*Corynebacterium ulcerans* infection is emerging in humans. We conducted phylogenetic analyses of *C. ulcerans* and *C. diptheriae*, which revealed diverse diphtheria toxin in *C. ulcerans*. Diphtheria toxin diversification could decrease effectiveness of diphtheria toxoid vaccine and diphtheria antitoxin for preventing and treating illnesses caused by this bacterium.

*Corynebacterium ulcerans* is a rod-shaped, aerobic, gram-positive bacterium closely related to *C. diphtheria*. Some strains of *C. ulcerans* can produce diphtheria toxin, which causes respiratory diphtheria in humans and animals. Reports of human infections with *C. ulcerans* have increased during the past 20 years, and *C. ulcerans* is a recognized emerging human pathogen (*1*). Humans can contract toxin-producing *C. ulcerans* from companion animals (*2*,*3*). Human death can occur if appropriate treatment is delayed (*4*). Non–toxin-producing *C. diphtheriae* and *C. ulcerans* can convert to toxin-producing strains through a process of lysogeny with diphtheria toxin gene–carrying corynebacteriophages (*5*–*7*). Although increased coverage of the diphtheria toxoid vaccine has reduced the frequency of *C. diphtheriae* infections, reports of *C. ulcerans* infections in humans are increasing.

A report evaluating the differences in the amino acid sequences of the diphtheria toxins in *C. diphtheriae* and *C. ulcerans* used only limited data, comparing 1 strain of *C. diphtheriae* against 2 strains of *C. ulcerans* (*8*), leaving the differences among the toxins of these 2 species unclear. Others have conducted bacterial genome analyses and deposited several genomic sequences of *C. diphtheriae* and *C. ulcerans* strains into a public database. We collected amino acid sequences of the diphtheria toxin and the nucleic acid sequences of the 16S rRNA gene of 6 *C. diphtheriae* strains and 6 *C. ulcerans* strains from the National Center for Biotechnology Information genome database (https://www.ncbi.nlm.nih.gov/genome). Then, we performed phylogenetic analyses by using MEGA 7.0 (https://www.megasoftware.net). 

We found that the 16S rRNA gene sequences divided into separate *C. diphtheriae* and *C. ulcerans* strains with some sequence variability among the strains in each species (Figure, panel A). The amino acid sequences of the toxins also divided into separate clades for each species. However, we noted that *C. diphtheriae* strains were identical, but *C. ulcerans* strains were diverse (Figure, panel B), suggesting that *C. ulcerans* tends to acquire mutations more frequently than *C. diphtheriae.* Two possible explanations for this phenomenon are that *C. ulcerans* is maintained by various animals, increasing its diversity compared with *C. diphtheria*, which is believed to infect only humans; or that *C. ulcerans* has a phage-independent pathway to acquire the diphtheria toxin–encoding gene, as reported (*9*). 

**Figure:**
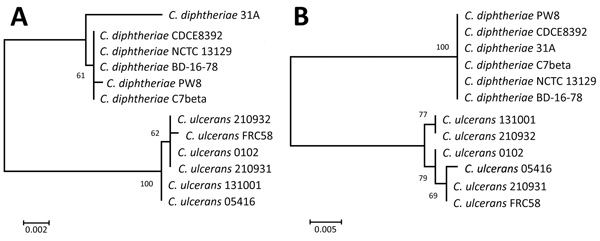
Phylogenetic analysis of the 16S rRNA gene sequences (A) and amino acid sequences (B) of diphtheria toxin genes of 6 *Coynebacterium ulcerans* strains and 6 *C. ulcerans* strains. All strains had the diphtheria toxin gene; whole-genome analysis data are available from the National Center for Biotechnology Information database (https://www.ncbi.nlm.nih.gov/genome). We generated phylogenetic trees by using the maximum-likelihood method in MEGA 7.0 (https://www.megasoftware.net). 16S rRNA gene sequences were analyzed by the Hasegawa-Kishino-Yano model with 1,000 bootstrap replications; amino acid sequences were analyzed by the Whelan and Goldman model with 100 bootstrap replications. Scale bars indicate substitutions per site.

Most severe human cases of disease caused by toxigenic *C. ulcerans* have occurred in unvaccinated or inadequately vaccinated persons. However, a fatal case was reported in a person who received a diphtheria vaccination booster ≈10 years before disease onset (*10*). Diversification of the *C. ulcerans* diphtheria toxin gene is of note because accumulation of these gene mutations potentially could lead to decreased effectiveness of the diphtheria toxoid vaccine for prevention and diphtheria antitoxin for treatment of toxigenic *C. ulcerans* disease.
